# Mitochonic acid 5 mitigates age-related hearing loss progression by targeting defective 2-methylthiolation in mitochondrial transfer RNAs

**DOI:** 10.3389/fncel.2025.1541347

**Published:** 2025-04-07

**Authors:** Teppei Kouga, Toru Miwa, Fan-yan Wei, Kishiko Sunami, Kazuhito Tomizawa

**Affiliations:** ^1^Department of Otolaryngology, Graduate School of Medicine, Osaka Metropolitan University, Osaka, Japan; ^2^Department of Otolaryngology-Head and Neck Surgery, Kyoto University, Kyoto, Japan; ^3^Department of Otolaryngology, Teikyo University Hospital, Kawasaki, Japan; ^4^Department of Modomics Biology and Medicine, Institute of Development, Aging and Cancer, Tohoku University, Sendai, Japan; ^5^Department of Molecular Physiology, Faculty of Life Sciences, Kumamoto University, Kumamoto, Japan

**Keywords:** age-related hearing loss, mitochonic acid 5, mitochondrial dysfunction, cyclin-dependent kinase 5 regulatory subunit-associated protein 1, 2-methylthiolation

## Abstract

**Introduction:**

Age-related hearing loss (ARHL) is linked to dementia, with mitochondrial dysfunction playing a key role in its progression. Deficient mitochondrial tRNA modifications impair protein synthesis and energy metabolism, accelerating ARHL. Mitochonic acid 5 (MA-5) has shown promise as a therapeutic candidate by improving mitochondrial function, reducing oxidative stress, and stabilizing membrane potential.

**Methods:**

In this study, we investigated the effects of MA-5 on ARHL in cyclin-dependent kinase 5 regulatory subunit-associated protein 1 (*Cdk5rap1*) knockout (KO) mice, which exhibit early-onset ARHL due to abnormalities in mitochondrial transfer RNA (mt-tRNA) modifications.

**Results:**

MA-5 treatment effectively attenuated ARHL progression in *Cdk5rap1*-KO mice by improving auditory brainstem response thresholds and distortion product otoacoustic emissions. It also reduced spiral ganglion and outer hair cell loss, while preserving the cochlear structural integrity by preventing mitochondrial degeneration in spiral ligament fibrocytes. Mechanistically, MA-5 upregulated the expression of silent information regulator sirtuin 1 and promoted the nuclear translocation of yes-associated protein, both of which are involved in regulating mitochondrial function and cellular senescence. Metabolomics analysis further demonstrated that MA-5 restored mitochondrial metabolism, reduced lactate accumulation, and maintained mitochondrial integrity.

**Conclusion:**

These findings suggest that MA-5 is a viable treatment option for ARHL and other age-related disorders associated with mitochondrial dysfunction.

## 1 Introduction

Aging is emerging as a major global health challenge. Japan, in particular, is experiencing an unprecedented rate of population aging, leading to substantial medical and social challenges. One of the most serious health concerns is age-related hearing loss (ARHL), which is closely associated with an increased risk of dementia (Uchida et al., 2018, 2022). Existing interventions, including hearing aids and cochlear implants, present significant financial and physical challenges, underscoring the urgent need for preventative strategies and novel treatments targeting the underlying causes of ARHL.

Despite extensive research on the pathophysiology of ARHL (Yang et al., 2023), its complex etiology and mechanisms of progression remain major impediments to the development of effective therapeutics. Mitochondria play a crucial role in cellular energy production, and mitochondrial dysfunction has been strongly implicated in aging. The development and progression of ARHL is significantly influenced by mitochondrial dysfunction, including the accumulation of mitochondrial DNA (mtDNA) damage, redox imbalances, oxidative stress-induced generation of reactive oxygen species (ROS), and impaired antioxidant function (Seidman et al., 1997; Pickles, 2003; Yamasoba et al., 2013; Kim et al., 2019).

Recent data highlight the critical role of precise mitochondrial ribosome translation in maintaining cytoplasmic proteostasis and nuclear gene expression, both of which collectively influence the cellular lifespan (Suhm et al., 2018). Defective chemical modifications of mitochondrial transfer RNAs (mt-tRNAs) have been associated with various human diseases, including type-2 diabetes, mitochondrial disorders, and normal aging (Yasukawa et al., 2001; Steinthorsdottir et al., 2007; Arragain et al., 2010; Suzuki et al., 2011; Torres et al., 2014; Karaiskos and Grigoriev, 2016; Steffen and Dillin, 2016). One such modification, 2-methylthio (ms^2^), is catalyzed by nuclear-encoded enzymes, including cyclin-dependent kinase (CDK)5 regulatory subunit-associated protein 1 (CDK5RAP1) (Arragain et al., 2010; Wei et al., 2011). The ms^2^ modification is a post-transcriptional modification of specific bases in mitochondrial tRNA, essential for accurate and efficient mitochondrial translation. Without this modification, codon-anticodon pairing is impaired, reducing the efficiency of mitochondrial ribosomal protein synthesis. The absence of ms^2^ modification disrupts mitochondrial protein synthesis, particularly affecting components of the electron transport chain, such as complexes I and IV. Consequently, mitochondrial oxidative phosphorylation is impaired, leading to insufficient ATP production. Under stress conditions, deficient ms^2^ modification significantly impairs mitochondrial protein synthesis, resulting in respiratory dysfunction and increased susceptibility to mitochondrial remodeling in *Cdk5rap1*-knockout (KO) mice (Wei et al., 2015). Our group previously demonstrated that CDK5RAP1 is crucial for mt-tRNA ms^2^ modifications for preserving mitochondrial function in the inner ear under stress-related conditions, such as aging-induced oxidative stress. Furthermore, *Cdk5rap1-*KO mice on a C57BL/6 background exhibit early-onset ARHL (Miwa et al., 2022, 2023), characterized by spiral ligament degeneration, reduced endocochlear potential, and hair cell loss. This early cochlear dysfunction is linked to elevated oxidative stress and cellular senescence. Moreover, mitochondrial disruption in fibrocytes of the spiral ligament exacerbates cochlear degeneration. These findings suggest that deficient tRNA modifications accelerate mitochondrial dysfunction and cochlear aging, providing insights into mitochondrial contributions to ARHL pathogenesis (Miwa et al., 2022, 2023). A deficiency in ms^2^ modification impairs mitochondrial protein synthesis, decreases energy metabolism, and increases ROS production, ultimately compromising mitochondrial remodeling under stress conditions. This mechanism contributes to early cochlear dysfunction and may play a key role in the progression of ARHL and other mitochondrial diseases.

Various therapeutic approaches, including gene therapy (Nourbakhsh et al., 2021), antioxidant administration (Miwa, 2021), caloric restriction (Someya et al., 2010), and plasma exchange (Mitani et al., 2023), have been explored for ARHL treatment. However, their ability to directly target mitochondrial dysfunction is limited, which has impeded their widespread clinical adoption.

Researchers at Tohoku University recently developed mitochonic acid 5 (MA-5), a novel compound derived from endogenous indole molecules. MA-5 has shown to enhance intracellular ATP production by specifically targeting mitochondrial structures, thereby improving mitochondrial function (Suzuki et al., 2015; Matsuhashi et al., 2017). This compound has been shown to boost mitochondrial energy production, increase respiration, and scavenge ROS, through activation of the mitogen-activated protein kinase (MAPK)–yes-associated protein (YAP) pathway (Lei et al., 2018). Unlike other therapeutic approaches, MA-5 directly targets mitochondrial dysfunction, positioning it as a promising candidate for treating ARHL. In *Cdk5rap1*-KO mice, defective ms^2^ of mitochondrial tRNAs leads to impaired mitochondrial translation, respiratory dysfunction, increased oxidative stress, and susceptibility to cochlear degeneration. Since MA-5 has been shown to enhance mitochondrial function by promoting mitochondrial biogenesis, reducing oxidative stress, and stabilizing mitochondrial membrane potential, it may hold therapeutic potential in *Cdk5rap1*-KO mice. Notably, MA-5 stabilizes the mitochondrial membrane potential and supports the assembly of respiratory chain complexes. Although the ms^2^ modification defect in KO mice impairs mitochondrial protein synthesis, improving mitochondrial membrane stability and respiratory complex activity could improve residual oxidative phosphorylation capacity, thereby supporting cellular energy demands and potentially mitigating cochlear degeneration.

In this study, we evaluated the effects of MA-5 administration on auditory function in mice with inner ear ATP depletion caused by impaired mt-tRNA modifications, aiming to determine whether MA-5 could suppress ARHL progression and mitigate its associated consequences.

## 2 Methods

### 2.1 Animals

CDK5RAP1-deficient mice (female, 64 weeks old, C57BL/6 J background) were generously provided by K. Tomizawa (Kumamoto University, Kumamoto, Japan). The *Cdk5rap1* knockout (KO) mice were generated by mating transgenic mice carrying floxed exons 5 and 6 of the *Cdk5rap1* with transgenic mice that express Cre recombinase under the control of the CAG promoter. Comprehensive genotyping information is available upon request. For all experiments, 12-week-old KO female mice (*n* = 99) and wild-type (WT) female mice (*n* = 25) were used. Both ears from each animal were included in the assessments. The sample size (n) represents the number of animals used. To clarify the distribution of animals across experiments following treatment and euthanasia, details are provided in Supplementary Table 1. MA-5 purchased from MedKoo Biosciences, Inc. (Durham, NC, United States) was administered orally through drinking water at concentrations of 2 mg/kg or 4 mg/kg daily for 2 months, starting at week 12 and continuing until week 20. The amount of water intake was monitored every 3 days, and adjustments were made to ensure equal consumption across groups. KO mice were randomly assigned to three groups receiving either vehicle (control; dimethyl sulfoxide [DMSO]), 2 mg/kg MA-5, or 4 mg/kg MA-5. Mean body weights were matched across all treatment groups at the start of experiment. The animals were housed under controlled conditions (25°C, 50% humidity) with ad libitum access to a standard diet (CE-2), and either MA-5-treated or normal drinking water. Fresh MA-5 solutions were prepared bi-weekly. After 8 weeks of treatment, the mice were euthanized via CO_2_ inhalation.

### 2.2 Study approval

All animal experiments were performed in compliance with established guidelines for the care and use of animals in neuroscience research. The study was approved by the Osaka Metropolitan University’s Committee on Use and Care of Animals (animal protocol number: 22035).

### 2.3 Auditory brainstem-response (ABR) analysis

Mice (*n* = 5/group) were anesthetized via intraperitoneal injection of a cocktail containing 0.3 mg/kg of medetomidine, 4.0 mg/kg of midazolam, and 5.0 mg/kg of butorphanol. Auditory brainstem-response (ABR) signals were recorded using three subcutaneous stainless-steel needle electrodes: the ground electrode was positioned close to the tail, and the positive and negative electrodes were positioned on the vertex and pinna, respectively. A NI USB-6216 signal processor (National Instruments, Austin, TX, United States) was employed to generate auditory stimuli consisting of 1-ms tone bursts with a 0.2-ms rise and fall time at a frequency of 14/s. An MF-1 magnetic speaker (Tucker-Davis Technologies, Alachua, FL, United States) was inserted into the ear canal to provide auditory stimuli within a restricted sound field. The ABR responses were band-pass filtered between 0.2 and 2 kHz, digitally sampled at 10 kHz, and then amplified 20,000 times. Each ABR waveform was averaged over 500 individual responses. The ABR threshold was defined as the lowest sound intensity that elicited a discernible response. Data were collected at stimulus intensities ranging from 0 to 90 dB sound pressure level (SPL) in 10 dB increments for frequencies of 4, 8, 16, and 32 kHz, and the threshold determination was performed via visual inspection of waveforms (Takeda et al., 2016; Miwa et al., 2019, 2023). For amplitude (μV) analysis, the peak amplitude (wave I) of the ABR signal was measured at each frequency. The analysis was performed offline in MATLAB (MathWorks, Natick, MA, United States) by setting the cursor to the maximum (peak) and minimum values (valleys) values of the wave. The average value of the upward and downward slopes (ΔV) of the peak was calculated as the amplitude.

### 2.4 Distortion product otoacoustic emissions (DPOAE) measurements

Distortion product otoacoustic emissions (DPOAE) measurements were conducted as described previously (Miwa et al., 2020, 2021). Briefly, animals were anesthetized, and their pinnae were removed to facilitate precise sound delivery. An ER10B+ probe microphone/speaker system (Etymotic Research, Inc., Elk Grove Village, IL, United States) with two speaker ports was securely fitted into the ear canal and connected to two closed-field MF-1 speakers (Tucker-Davis Technologies, Alachua, FL, USA). Two primary tones were generated (1-s duration with a 20-ms rise/fall cosine ramp; f₂/f₁ = 1.22, with f₂ varying at one-fourth octave steps from 4 to 20 kHz) and separately routed to the MF-1 speakers at SPL of SPL₁ = 75 dB and SPL₂ = 65 dB. Responses were digitized at 150 kHz using a NI USB-6216 signal processor with an analog-to-digital converter (National Instruments), amplified by 20 dB, and DPOAE were analyzed using customized software written in LabVIEW (National Instruments). Each recording was repeated 10 times at 20-s intervals, and averaged as a function of time (*n* = 5/group). Noise levels were estimated by averaging three adjacent frequency bins above and below the DPOAE frequency.

### 2.5 Endocochlear potential (EP) measurements

Endocochlear potential (EP) values were measured in anesthetized mice by surgically exposing the cochlea and creating a small opening in the spiral ligament (SLi) to access the scala media (SM) within the middle turn of cochlea. A heat-pulled micropipette electrode filled with 150 mM KCl was carefully inserted into the SM, and stable potentials were recorded using an MEZ-7200 amplifier (Nihon Koden, Tokyo, Japan) (n = 5 mice/group) (Miwa et al., 2019, 2020).

### 2.6 Tissue processing

At 20 weeks of age, cochleae were harvested from control and MA-5-treated KO mice, fixed in 4% paraformaldehyde for 12 h at 4°C, and decalcified in 0.5 mol/L ethylenediaminetetraacetic acid at 25°C for 3 days. The fixed tissues were embedded in an optimal cutting temperature medium with an and cryosectioned into 12 μm-thick slices for further analysis.

### 2.7 Hematoxylin and eosin (H&E) staining and measurement of the width of the stria vascularis

Sections containing the midmodiolar region were stained using hematoxylin and eosin (H&E) for assessing the gross morphology of cochlear tissues. Images were captured using a BZ-9000 microscope (Keyence, Osaka, Japan). Five sections were randomly selected around the central long axis of the modiolus per cochlea (*n* = 5/group). Additionally, the images were used to measure the width of the stria vascularis, with a mean and standard error (SE) calculated for each genotype, as previously described (Shibata et al., 2016).

### 2.8 Immunohistochemical staining

Sections were incubated with primary antibodies: anti-Na^+^/K^+^-ATPase α1 (1:200), anti-connexin 26 (Cx26; 1:200), anti-silent information regulator sirtuin 1 (SIRT1; 1:200), and anti-YAP (1:100). After incubation, the sections were blocked with 10% goat serum and incubated for 1 h at 25°C, incubated with primary antibodies and treated with Alexa Fluor 594- or Alexa Fluor 488-conjugated secondary antibodies for counterstaining. Nuclear counterstaining was performed using Hoechst 33258 dye. Images were captured using a BZ-9000 fluorescence microscope. Five sections were randomly selected around the central long axis of the modiolus per cochlea (*n* = 5/group).

### 2.9 Modified labeling index (mLI) analysis

As previously reported (Takeda et al., 2016; Miwa et al., 2023), immunostaining levels in cryosectioned cochlear samples were quantitatively assessed using the modified labeling index (mLI). Briefly, images were captured at 1,360 × 1,024 pixels using identical exposure settings across all groups. The Magic Wand tool in Adobe Photoshop (Adobe Systems, San Jose, CA, United States) was used to select background tissue-free regions outside the cochlear portions. The tolerance level was adjusted to accurately define these regions for background intensity measurements. Images were converted to 16-bit grayscale, and stained regions were selected using the same method as background selection. The Histogram tool in Photoshop was used to determine the optical densities of both the background and stained regions. The staining intensity of the region of interest was subtracted from the mean background intensity to determine the final staining intensity. The percentage of stained pixels to the total pixels in the image was used to compute staining ratio. Blinding was applied to all mLI measurements (*n* = 5/group).

### 2.10 Hair cell and spiral ganglion cells counts

To assess hair cell integrity, cochleae were stained with Texas Red-X phalloidin to visualize F-actin expression and imaged using a fluorescence microscope. Inner hair cells (IHCs) and outer hair cells (OHCs) were counted along 140-μm basal segments of the basilar membrane (*n* = 5/group) (Miwa et al., 2013, 2019, 2020). Spiral ganglion cells (SGCs) were visualized via immunohistochemical staining using anti-beta-tubulin III (Tuj1) antibodies and positively stained cells were counted in Rosenthal’s canal (*n* = 5/group) (Yamada et al., 2014; Miwa et al., 2020).

### 2.11 Quantitative assessment of senescence-associated *β*-galactosidase expression

Senescent cells were detected using a senescence-associated β-galactosidase (SA-βgal) Kit (Cosmo Bio Co., Ltd., Tokyo, Japan). The Cell Profiler program was utilized to analyze the images, and quantification was conducted in a blinded manner (*n* = 5/group).

### 2.12 Enzyme-linked immunosorbent assay (ELISA)

Mice were sacrificed by cervical dislocation, and the inner ears were carefully dissected and stored at −80°C until analysis. Cochlear protein lysates were prepared by homogenizing tissue samples using a Sonifier S-250A ultrasonic processor (Branson, Danbury, CT, United States). Protein levels were quantified using a Mouse SIRT1 ELISA kit (Abcam, Cambridge, UK), following the manufacturer’s instructions. Absorbance was measured at 450 nm using a Multiskan FC microplate reader (Thermo Fisher Scientific, Waltham, MA, United States) (*n* = 5/group).

### 2.13 Western blot analysis

The same lysates used for ELISA were subjected to 12.5% sodium dodecyl sulfate-polyacrylamide gel electrophoresis (SDS-PAGE). Proteins were detected using primary antibodies against YAP (1:1,000) and horseradish peroxidase (HRP)-conjugated secondary antibodies (Bio-Rad, Hercules, CA, United States). For loading control detection, HRP-conjugated anti-*β*-actin antibody (PM053-7; MBL, Nagoya, Japan) was applied at a dilution of 1:5,000. Protein bands were visualized using an enhanced chemiluminescence system (Bio-Rad) and analyzed with ImageJ software (NIH). β-actin served as the internal control for normalization (*n* = 5/group).

### 2.14 Transmission electron microscopy (TEM) analysis

Cochleae were embedded for ultrathin sectioning following fixation, post-fixation with osmium tetroxide, staining with uranyl acetate, and dehydration. Sections were examined using a Talos F200C Transmission Electron Microscope (TEM; FEI, Tokyo, Japan) to assess mitochondrial morphology and damage (*n* = 5/group). We evaluated mitochondrial size and damage using previously reported methods (Miwa et al., 2023). In brief, for accurate representation, 20 images of each sample were captured at the same plane and magnification. The quantity of damaged and normal mitochondria in each image was assessed using the mitochondrial crista score, which reflects structural abnormalities, in ImageJ software (National Institutes of Health, Bethesda, MD, United States). The ratio of damaged to normal mitochondria was then calculated. Additionally, the dimensions of elongated mitochondria in each image were measured (*n* = 5 for each group). The outer membrane of each mitochondrion was traced using the freehand tool in ImageJ software (National Institutes of Health).

### 2.15 Metabolome analysis

Cochlear metabolites were extracted and analyzed using liquid chromatography-mass spectrometry (LCMS-8060NX, Shimadzu Co., Tokyo, Japan). Peaks were detected and analyzed using Travers MS software (Reifycs Inc., Tokyo, Japan). The data were further processed using MetaboAnalyst^1^ for principal component analysis (PCA), heat map analysis, enrichment analysis, pathway analysis, and network analysis (*n* = 3/group). In metabolome analysis, PCA is a dimensionality reduction technique that transforms high-dimensional data into lower dimensions, facilitating visualization of sample similarities and differences to elucidate group separation and identify characteristic metabolic patterns. Heatmap analysis complemented this by offering a visual representation of metabolite concentration changes through color gradients, providing critical insights into metabolite variability and clustering across samples. Enrichment analysis was performed to statistically assesses whether specific metabolite sets were significantly associated with particular biological pathways or functions, highlighting key biological processes. Pathway analysis identified relevant metabolic pathways involving fluctuating metabolites and evaluated their roles and variations within these pathways. Network analysis, on the other hand, constructed a visual framework of interactions among metabolites, genes, and proteins, enabling a holistic understanding of the metabolic network and pinpointing pivotal hub metabolites. The integration of these analytical approaches allowed for a comprehensive and robust biological interpretation of metabolome data.

### 2.16 Nuclear:cytoplasmic ratio calculations

The nuclear:cytoplasmic ratio (NCR) of the YAP protein was calculated using immunohistochemical images obtained with the anti-YAP antibody, as described in section 2.8. This approach was employed to detect subtle subcellular changes in small cell populations. Fluorophore-intensity measurements were obtained and analyzed using partial differential equation simulation application.^2^

### 2.17 Statistical analyses

Data were analyzed using one-way or two-way analysis of variance followed by post-hoc Tukey’s test, performed with GraphPad Prism Ver. 10.0.0 for Windows (GraphPad Software, La Jolla, CA, United States). Statistical power and sample sizes were determined using ‘PS: Power and Sample Size Calculation’ software (Department of Biostatistics, Vanderbilt University, Nashville, TN, United States) (Dupont and Plummer, 1990). Data were presented as mean ± SE. The threshold for statistical significance was set at *p* < 0.05.

## 3 Results

### 3.1 General

All experiments were performed using *Cdk5rap1*-KO mice, which were treated with either a low (2 mg/kg daily) or high (4 mg/kg daily) concentration of MA-5 or with a vehicle (DMSO; untreated or control), as described in the Methods section. We used two concentrations to investigate the drug’s dosage dependency.

### 3.2 Treatment with a low concentration of MA-5 attenuated ARHL progression

**Figure 1:**
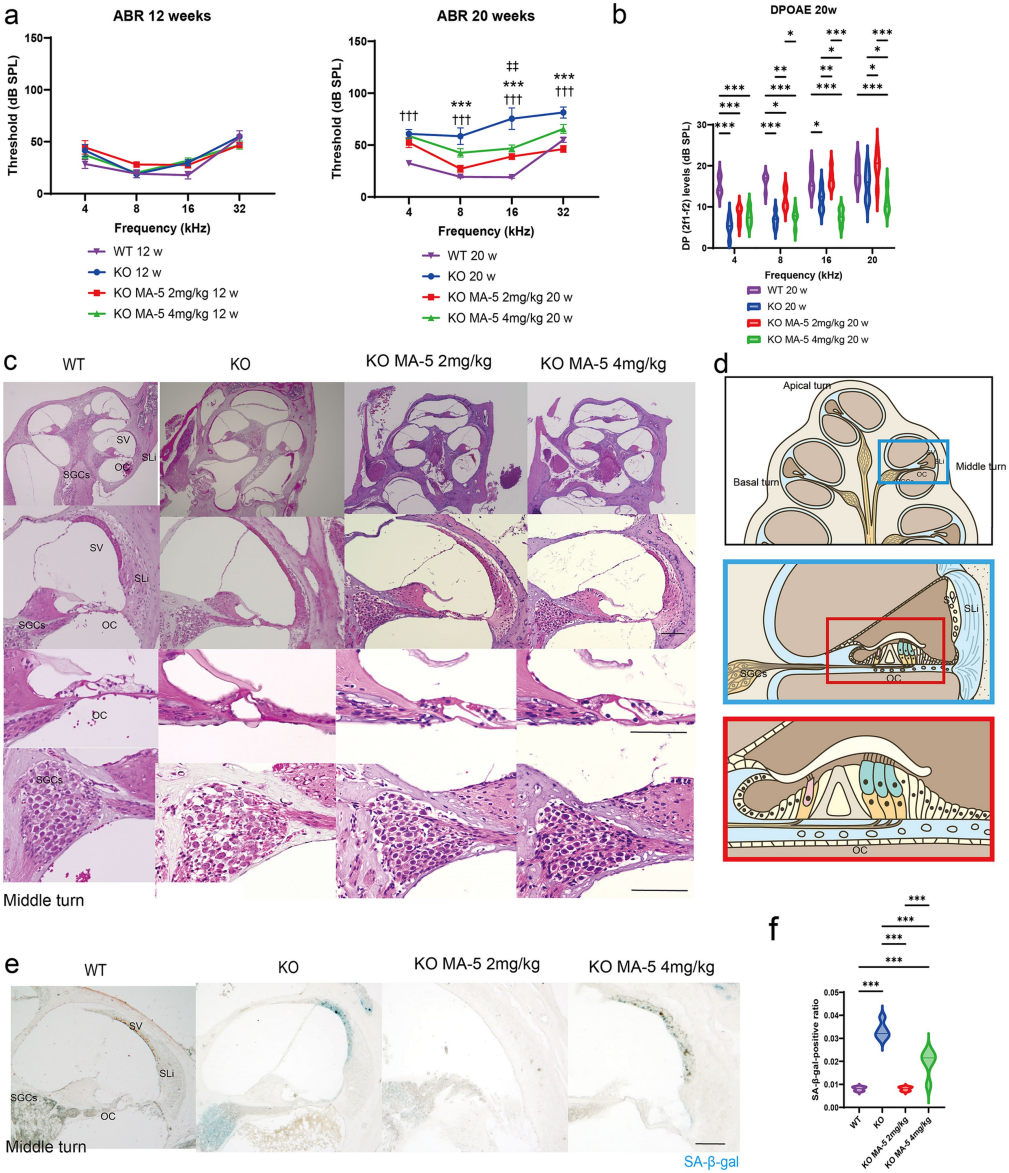
MA-5 ameliorated ARHL and cochlear senescence in *Cdk5rap1*-KO mice. **(a)** ABR thresholds in *Cdk5rap1*-KO untreated mice and WT untreated mice at 12 and 20 weeks of age compared to those in MA-5-treated mice across various frequencies (*n* = 5 mice/group). Error bars represent the mean ± SE of five independent experiments. ^†††^ indicates the significant differences (*p* < 0.001) between WT mice vs. untreated KO mice. *** indicates significant differences (*p* < 0.001) between untreated KO mice and MA-5 treated mice (2 mg/kg). ^‡‡^ indicates significant differences (*p* < 0.01) between untreated KO mice and MA-5 treated mice (4 mg/kg). **(b)** DPOAE levels in *Cdk5rap1*-KO untreated mice and WT untreated mice at 20 weeks of age compared to those in MA-5-treated mice across various frequencies (*n* = 5 mice/group). Error bars represent the mean ± SE of five independent experiments. *p < 0.05, **p < 0.01, ***p < 0.001. **(c)** Gross cochlear morphology, including the organ of Corti (OC), spiral ganglion cells (SGCs), stria vascularis (SV), and spiral ligament (SLi), at 20 weeks of age (*n* = 5 /group). **(d)** Cartoon diagram of cross-section of the mouse cochlea highlighting key structures. The blue and red boxes represent the middle turn of the cochlea and a zoomed-in view of the OC, respectively. **(e)** SA-*β*-gal staining of cochlear sections from the middle turn at 20 weeks of age, showing senescent cells in blue (*n* = 5/group). **(f)** Quantification of SA-β-gal-positive cells in the cochlea (*n* = 5/group). ***p < 0.001. Scale bars = 100 μm. MA-5, mitochonic acid 5; cdk5rap1, cyclin-dependent kinase (CDK)5 regulatory subunit-associated protein 1; KO, knockout; SA-β-gal, senescence-associated *β*-galactosidase; ARHL, age-related hearing loss; ABR, auditory brainstem-response; DPOAE, distortion product otoacoustic emissions; SLi, spiral ligament; SV, stria vascularis; SGCs; spiral ganglion cells; OC, Organ of Corti; SE, standard error of mean.

ABR thresholds did not differ significantly between 12-week-old *Cdk5rap1*-KO mice and WT mice across all tested frequencies (Figure 1a). At 20 weeks, untreated KO mice exhibited significantly higher ABR thresholds than WT mice at all frequencies (Figure 1a; all *p* < 0.001; *F* (3, 64) = 21.27, *p* = 0.004). The ABR thresholds were significantly lower at 8, 16, and 32 kHz in low-concentration MA-5-treated KO mice than in untreated ones (Figure 1a; *p* < 0.001 for all; *F* (3, 64) = 21.27, *p* = 0.004). Furthermore, at 8 and 32 kHz, the progression of hearing loss was suppressed to the extent that there was almost no difference from WT. Although treatment with a high concentration of MA-5 (4 mg/kg daily) also suppressed the progression of hearing loss, it was not completely effective (Figure 1a; 16 kHz: *p* < 0.001; *F* (3, 64) = 21.27, *p* = 0.004).

To assess auditory neuron synapse functionality, we measured the amplitude of ABR Wave I. A statistically significant difference was observed at 4 kHz between untreated KO mice and those treated with MA-5 at a dose of 2 mg/kg (Supplementary Figure 1; 90 dB; *p* = 0.01, 80 dB; *p* = 0.03, 70 dB; *p* = 0.007, *F* (24, 128) = 6.666, *p* < 0.001). No significant differences were detected among the other experimental groups (Supplementary Figure 1).

Similarly, untreated KO mice had significantly lower DP levels than WT mice (4, 8 kHz; *p* < 0.001, 16 kHz; *p* = 0.03, F (3, 64) = 31.29, *p* < 0.001). DP levels were significantly higher at 8, 16, and 20 kHz in the low concentration MA-5-treated mice compared to untreated KO mice, except at 4 kHz (Figure 1b: 8 kHz, *p* = 0.008; 16 kHz, *p* = 0.009; 20 kHz, *p* = 0.03; F (3, 64) = 31.29, *p* < 0.001). Compared to the DP level in WT, the difference was minimized to the extent that it was not recognized only at 16 and 20 kHz. In contrast, treatment with a higher concentration of MA-5 resulted in lower DP levels at 16 and 20 kHz than those found in the untreated mice (Figure 1b: 16 kHz, *p* = 0.04; 20 kHz, *p* = 0.04; F (3, 64) = 31.29, *p* < 0.001).

### 3.3 MA-5 treatment inhibited the loss of SGCs and fibrocytes in the SLi, with no change in SV

Cochlear morphology appeared normal in both untreated and MA-5-treated mice. However, untreated mice exhibited significant SGC losses, compared to those treated with MA-5 (Figures 1c,d). Similarly, fibrocyte loss in the SLi was evident in untreated mice but was not observed in the MA-5-treated group (Figures 1c,d). No significant morphological differences or changes in stria vascularis (SV) thickness were detected across all experimental groups (Figure 1c and Supplementary Figure 2).

### 3.4 Low concentration of MA-5 inhibited cochlear senescence

Senescent cells were detected in the SGC, SLi, and SV regions of the cochleae at 20 weeks in untreated KO mice (Figures 1d,e). The percentage of SA-*β*-gal-positive cells was significantly higher in untreated KO mice than in WT mice, whereas the low-concentration MA-5-treated group exhibited significantly lower SA-β-gal positivity compared to untreated mice (Figures 1f; *p* < 0.001; *F* (3, 16) = 60.34, *p* < 0.001). There was no difference in SA-β-gal positivity between the WT and low-concentration MA-5-treated groups. Although high-concentration MA-5 treatment also reduced SA-β-gal positivity compared to untreated KO mice (*p* < 0.001; *F* (3, 16) = 60.34, *p* < 0.001), the reduction was less pronounced than that observed in the low-concentration group (*p* = 0.002; *F* (3, 16) = 60.34, *p* < 0.001).

### 3.5 Low concentrations of MA-5 inhibited OHC loss, whereas a high concentration of MA-5 accelerated it

**Figure 2:**
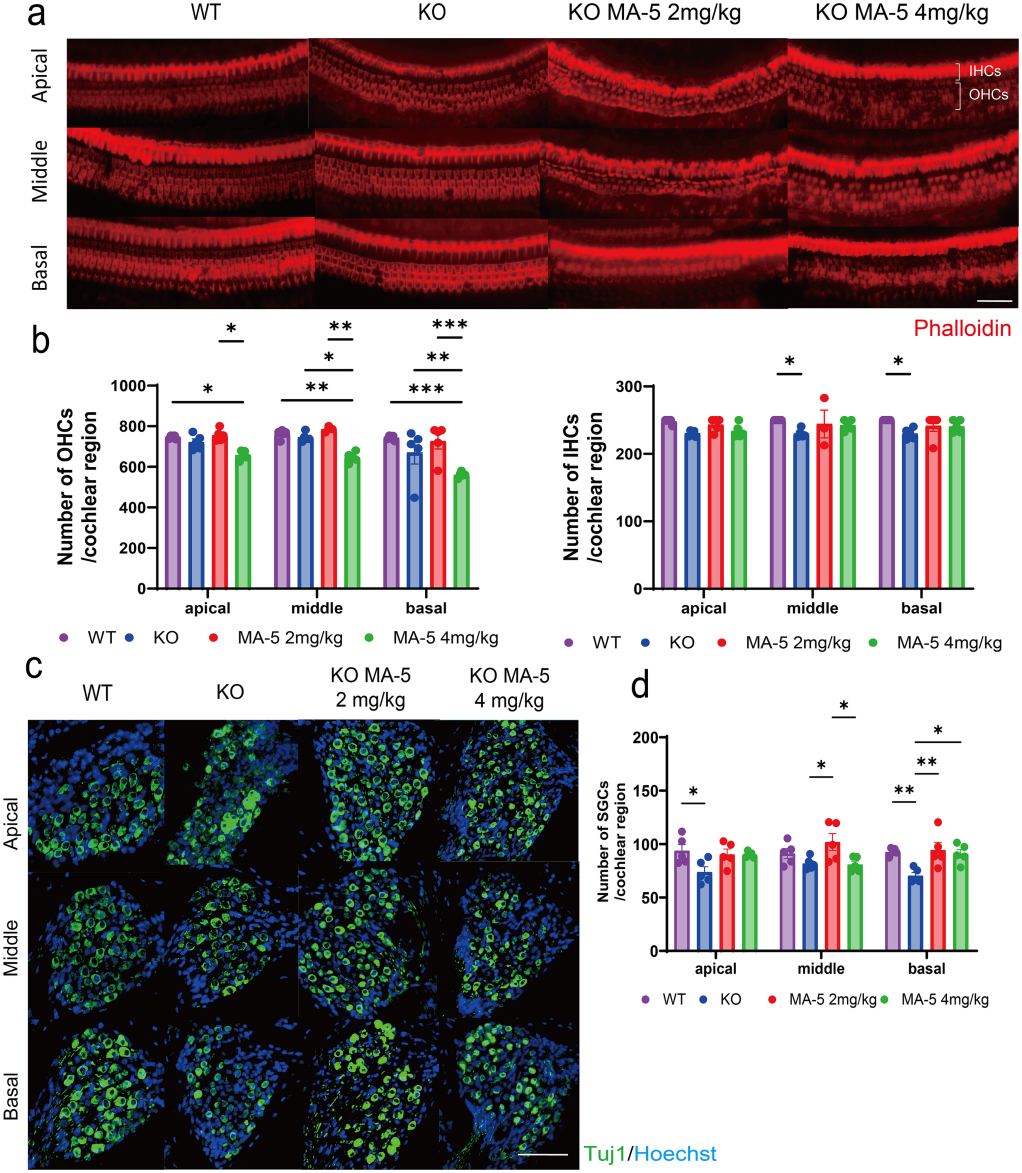
MA-5 partially prevented the loss of HCs and SGCs in *Cdk5rap1*-KO mice. **(a)** Phalloidin staining of IHCs and OHCs in untreated and MA-5-treated cochleae (*n* = 5/group). **(b)** Quantitative analysis of OHCs and IHCs (*n* = 5/group). **(c)** Anti-Tuj1 immunostaining of SGCs in cochleae from untreated and MA-5-treated mice (*n* = 5/group). **(d)** SGC counts in untreated and MA-5-treated mice (*n* = 5/group). *p < 0.05, **p < 0.01, ***p < 0.001. Scale bars = 100 μm. MA-5, mitochonic acid 5; cdk5rap1, cyclin-dependent kinase (CDK)5 regulatory subunit-associated protein 1; Tuj1, beta-tubulin III; KO, knockout; HC, hair cell; SGCs; spiral ganglion cells; IHCs, inner hair cells; OHCs, outer hair cells.

The number of OHC cells was reduced in both WT and untreated *Cdk5rap1*-KO mice, though the reduction not statistically significant. Comparison of OHC counts between untreated and MA-5-treated KO mice revealed no significant difference in the low-concentration MA-5-treated group. In contrast, the high-concentration MA-5-treated group exhibited significantly lower OHC counts compared to untreated mice (Figures 2a,b: middle, *p* = 0.02; basal, *p* = 0.006; *F* (2, 46) = 7.545, *p* = 0.001). Furthermore, the number of OHCs was also significantly reduced compared to WT mice (Figures 2a,b: apical, *p* = 0.03; middle, *p* = 0.004; basal, *p* = 0.004; *F*(2, 46) = 7.545, *p* = 0.001). The number of IHCs was found to be lower in KO mice than in WT mice (Figures 2a,b: middle, *p* = 0.04; basal, *p* = 0.04; F (2, 46) = 7.252, *p* = 0.04), but no significant difference was found between the untreated and MA-5 treated groups.

### 3.6 Low-concentration MA-5 inhibited SGC loss

Untreated KO mice showed significantly greater SGC loss than WT mice, particularly in the apical and basal cochlear regions (Figures 2c,d; apical rotation, *p* = 0.02; basal, *p* = 0.008; *F* (2, 48) = 10.8, *p* < 0.001). Mice treated with low-concentration MA-5 exhibited significantly less SGC loss than untreated KO mice (Figures 2c,d; middle panel, *p* = 0.02; basal, *p* = 0.004; *F* (2, 48) = 10.8, *p* < 0.001). The high concentration of MA-5 had a milder protective effect showing significant SGC preservation only in the basal turn of the cochlea (Figures 2c,d; *p* = 0.02; *F* (2, 48) = 10.8, *p* < 0.001). No significant differences were observed in other cochlear regions (Figures 2c,d; *p* = 0.01; *F* (2, 36) = 0.4466, *p* = 0.64).

### 3.7 MA-5 treatment mitigated EP decline and supported SLi function

**Figure 3:**
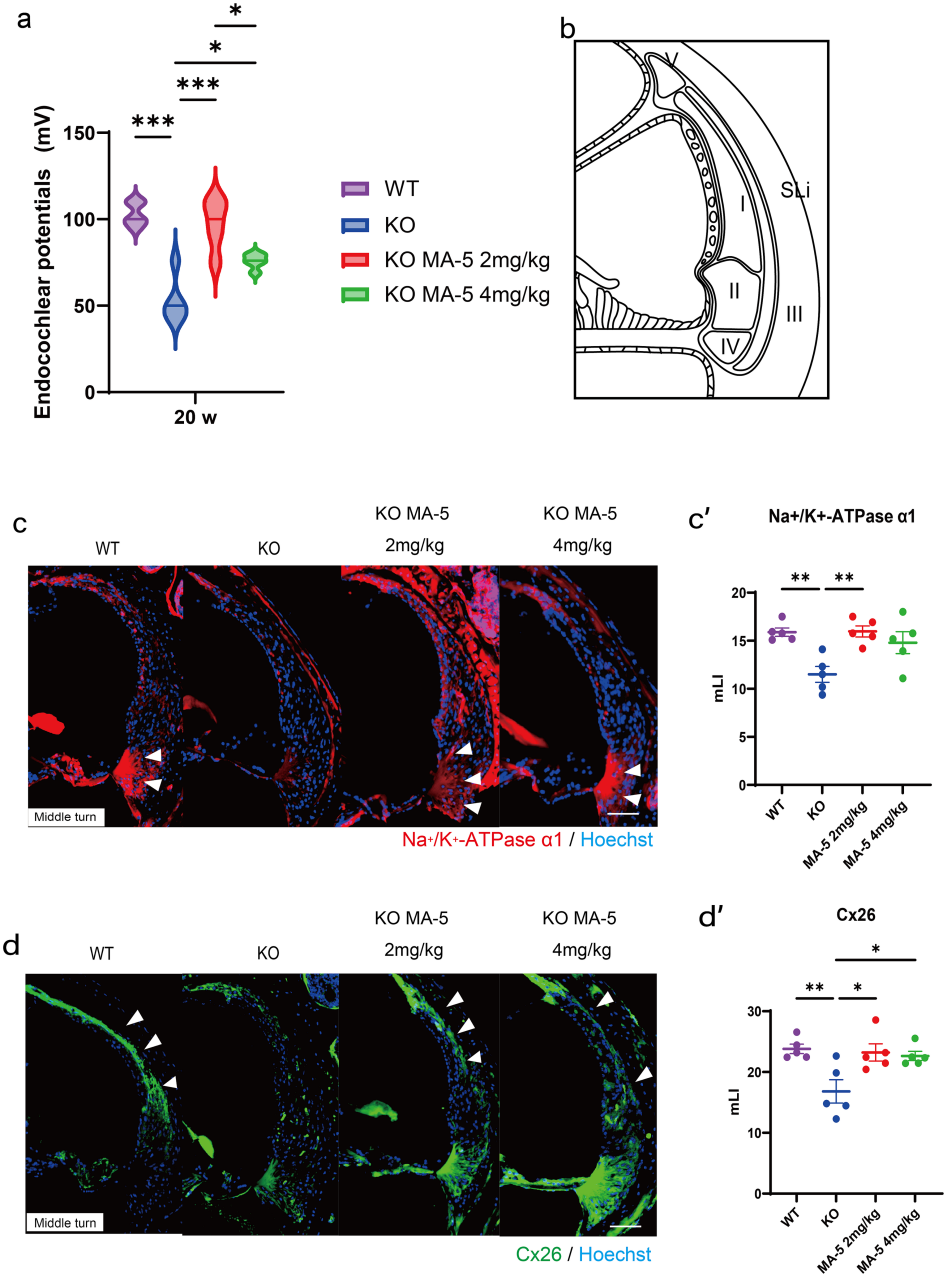
Endocochlear potential and immunohistological analysis of SLi tissues. **(a)** Endocochlear potential in *Cdk5rap1*-KO mice (untreated and MA-5-treated) and WT untreated mice at 20 weeks of age. **(b)** Schematic representation of the five different types of SLi fibrocytes. **(c)** Immunohistochemical staining of SLi tissues from untreated and MA-5-treated mice in the middle turn. Na^+^/K^+^-ATPase α1 expression (red) was detected as a marker of fibrocytes (types II, IV, and V). Arrowheads indicate Na^+^/K^+^-ATPase α1 expression. **(c′)** Quantitative analysis of Na^+^/K^+^-ATPase α1 expression in untreated and MA-5-treated mice, based on the mLI. **(d)** Immunohistochemical staining of SLi tissues showing Cx26 (green) expression in type-I fibrocytes in the middle turn. Arrowheads indicate Cx26 expression. **(d′)** mLI analysis of Cx26 expression in untreated and MA-5-treated mice. *p < 0.05, **p < 0.01, ***p < 0.001. Scale bars = 100 μm. MA-5, mitochonic acid 5; cdk5rap1, cyclin-dependent kinase (CDK)5 regulatory subunit-associated protein 1; KO, knockout; SLi, spiral ligament; mLI, modified labeling index; Cx26, connexin 26.

At 20 weeks of age, the EP was significantly reduced in untreated KO mice compared to WT mice (Figure 3a; *p* < 0.001, *F* (3, 16) = 22.19, *p* < 0.001). However, treatment with a low concentration of MA-5 (2 mg/kg) significantly preserved EP levels (97.2 ± 9.1 mV) compared to untreated mice (Figure 3a; *p* < 0.001; *F* (1, 24) = 41.98, *p* < 0.001). Treatment with a high concentration of MA-5 (4 mg/kg) also mitigated EP decline (79.8 ± 8.7 mV, *p* = 0.02; *F* (3, 16) = 22.19, *p* < 0.001) but the effect was less pronounced than that observed with the low-concentration treatment. Immunohistochemical staining revealed that Na^+^/K^+^-ATPase α1 expression in SLi tissues was substantially higher in low concentration-MA-5 treated mice than in untreated mice, reaching levels comparable to WT mice (Figures 3b,c,c′, *p* = 0.005; *F* (3, 16) = 7.009, *p* = 0.003). Similarly, Cx26 expression in SLi tissues was significantly elevated in low-concentration MA-5-treated mice, reaching levels comparable to WT mice (Figures 3b,d,d′, *p* = 0.02; *F* (3, 16) = 6.154, *p* = 0.006). A high concentration of MA-5 resulted only in a moderate increase in Cx26 expression (Figures 3d, d′, *p* = 0.03; *F* (3, 16) = 6.154, *p* = 0.006).

### 3.8 MA-5 treatment inhibited mitochondrial degeneration in SLi fibroblasts

**Figure 4:**
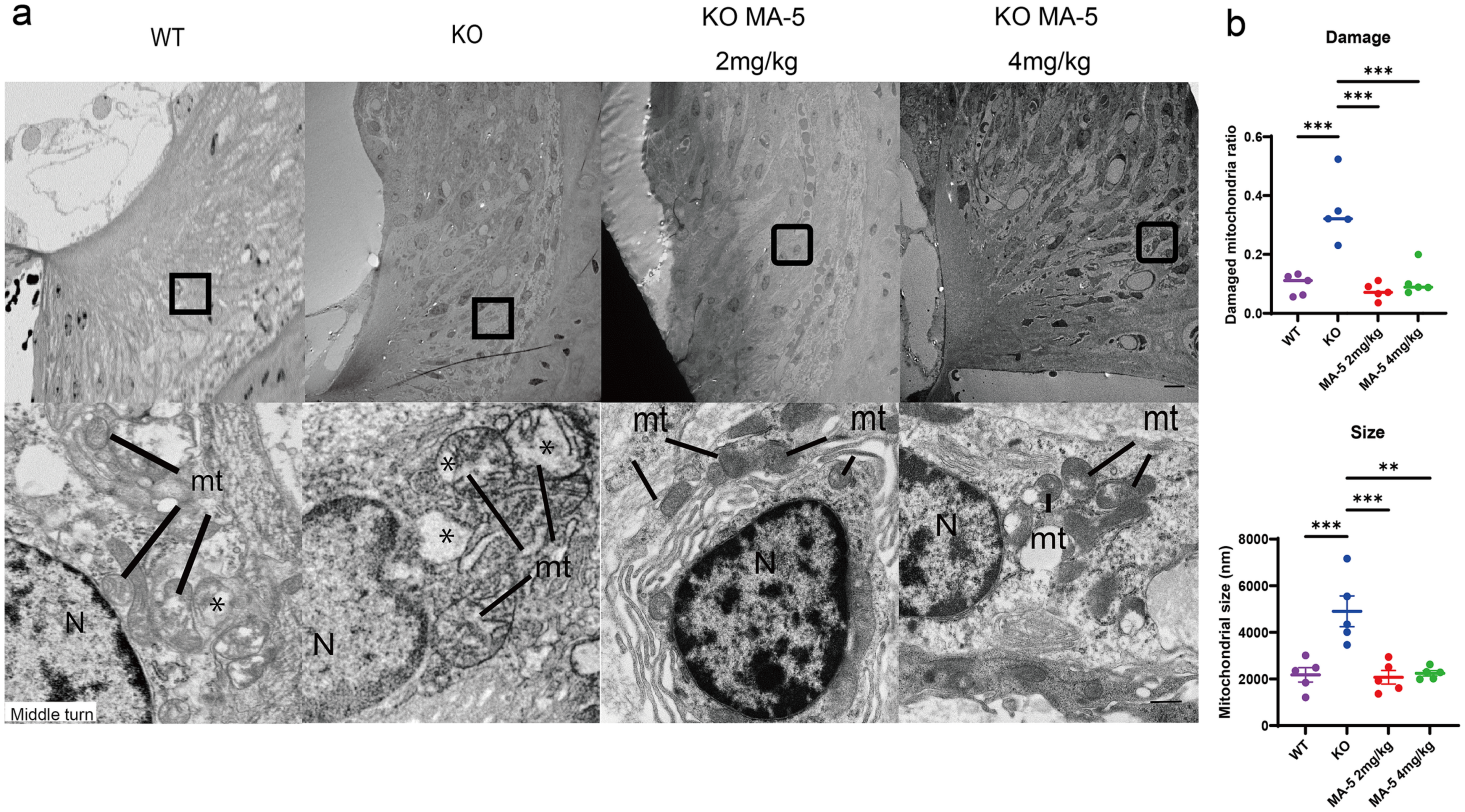
Restoration of type-II and type-IV fibrocytes in SLi mitochondria by MA-5 in *Cdk5rap1*-KO mice. **(a)** TEM images of type-II and type-IV fibrocytes from the middle turn of the cochlea. N = nucleus. Magnified views highlight mitochondrial cristae structures in *Cdk5rap1*-KO mice (untreated and MA-5-treated), with asterisks marking cristae loss. mt = mitochondria. Scale bars = 1 μm (full image) and 100 nm (magnified image). **(b)** Quantification of mitochondrial damage and size in untreated and MA-5-treated mice. **p < 0.01, ***p < 0.001. MA-5, mitochonic acid 5; cdk5rap1, cyclin-dependent kinase (CDK)5 regulatory subunit-associated protein 1; KO, knockout; SLi, spiral ligament; TEM, transmission electron microscopy.

TEM images showed that mitochondria in type-II and IV fibroblasts of the SLi from untreated KO mice displayed severe losses of cristae at 20 weeks of age, compared to WT mice. However, this mitochondrial degeneration was attenuated in MA-5-treated mice (Figure 4a). Mitochondrial damage was significantly lower in the MA-5-treated mice than in untreated mice, reaching levels comparable to WT mice (Figure 4b, both *p* < 0.001; *F* (3, 16) = 20.15, *p* < 0.001). Additionally, mitochondria were significantly smaller in MA-5-treated mice than in untreated ones, with sizes equivalent to those observed in WT mice (Figures 2, 4b mg/kg, *p* < 0.001; 4 mg/kg, *p* = 0.001; *F* (3, 16) = 12.02, *p* < 0.001).

### 3.9 MA-5 activated SIRT1 and promoted nuclear localization of YAP to inhibit senescence

**Figure 5:**
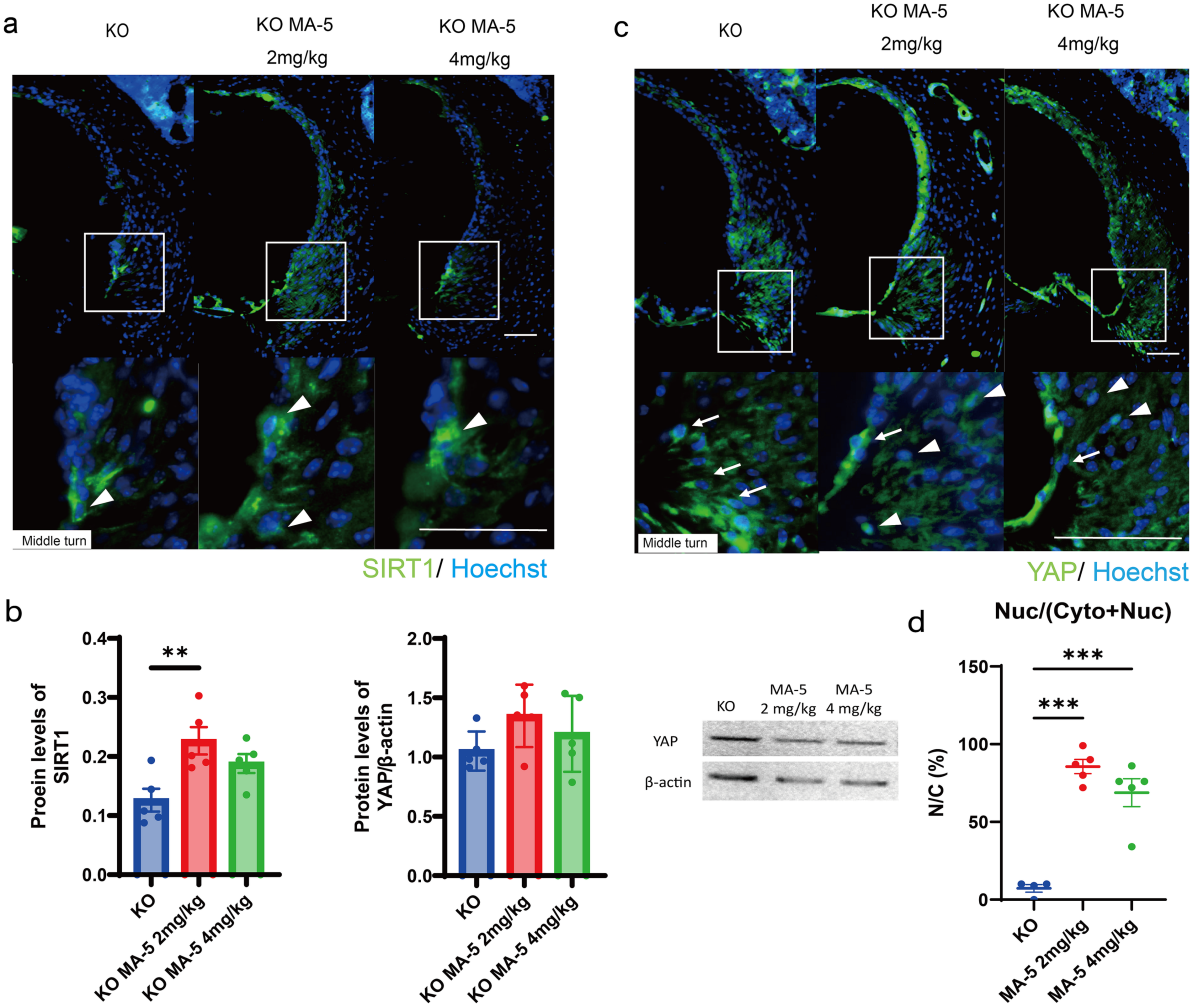
MA-5 modulated key molecular regulators in the SLi. **(a)** Immunohistochemical staining for SIRT1 (green) in SLi tissues from untreated and MA-5-treated mice. Arrowheads indicate SIRT1 expressing cells. **(b)** Quantitative analysis of SIRT1 expression (ELISA) and YAP expression (western blot) in untreated and MA-5-treated mice. **(c)** Immunohistochemical staining for YAP (green) in SLi tissues from untreated and MA-5-treated mice. Arrowheads indicate nuclear YAP, while arrows indicate cytoplasmic YAP expression. **(d)** Comparison of the NCRs of YAP in untreated and MA-5-treated mice. **p < 0.01, ***p < 0.001. Scale bars = 100 μm. MA-5, mitochonic acid 5; cdk5rap1, cyclin-dependent kinase (CDK)5 regulatory subunit-associated protein 1; KO, knockout; SLi, spiral ligament; SIRT1, silent information regulator sirtuin; ELISA, enzyme-linked immunosorbent assay; YAP, yes-associated protein; NCR, nuclear-to-cytoplasmic ratio.

SIRT1 expression, which was downregulated in untreated mice (Miwa et al., 2022), was significantly upregulated in the group treated with a low concentration of MA-5 and moderately upregulated in the high-concentration-treated group (Figure 5a). Quantitative ELISA confirmed that SIRT1 expression was significantly elevated in the low-concentration group (*p* = 0.01; Figures 5b,f (2, 12) = 6.562, *p* = 0.01). Although no variation in YAP expression was seen among the groups (Figures 5b,c), the nuclear localization of YAP was significantly higher in MA-5-treated mice than in untreated mice (Figures 5c,d; *p* < 0.001 for both; F (2, 12) = 49.37, *p* < 0.001).

### 3.10 MA-5 enhanced mitochondrial metabolism in SLi fibroblasts

**Figure 6:**
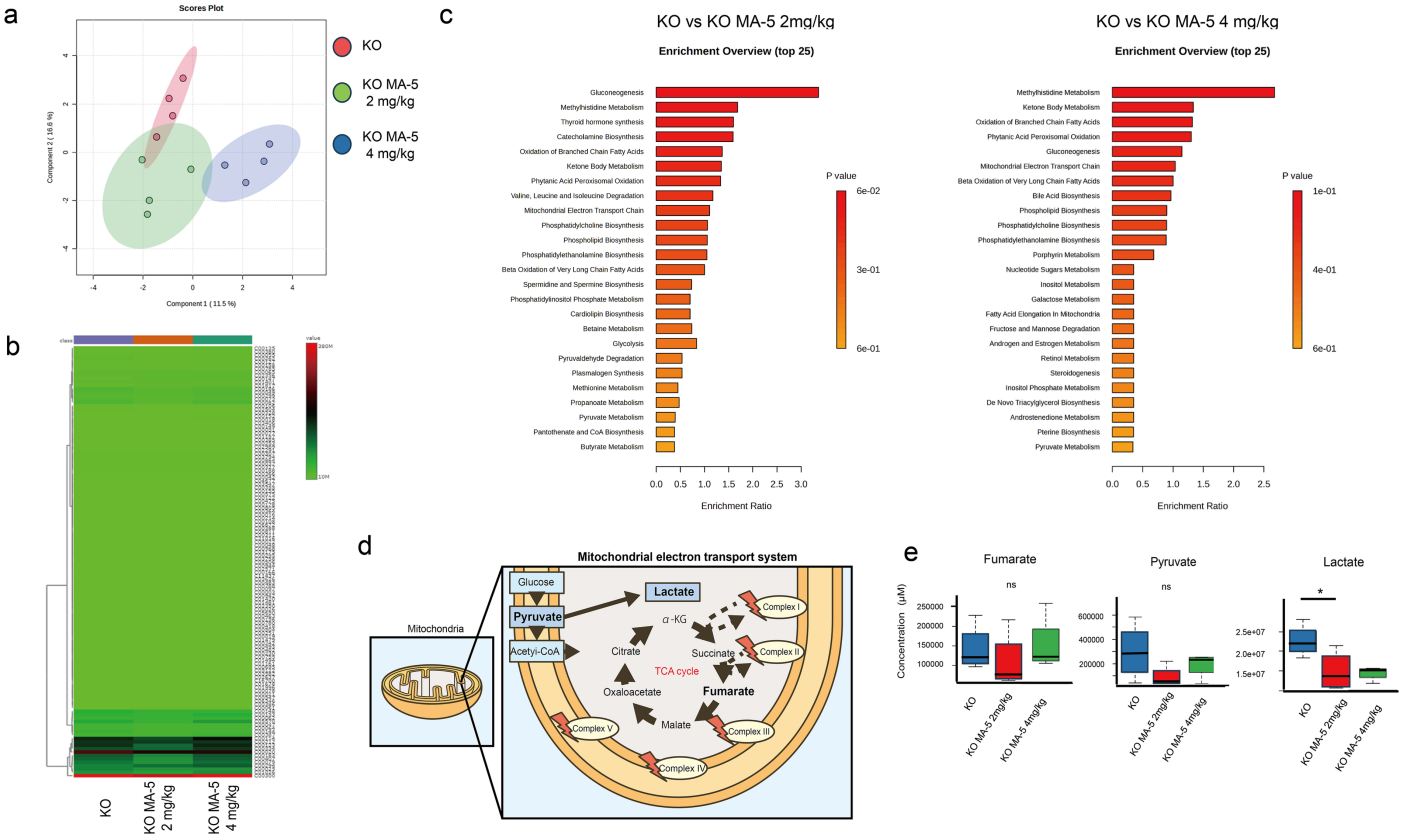
Mitochondrial metabolite changes in MA-5-treated *Cdk5rap1*-KO cochleae. **(a)** PCA of metabolome data (*n* = 3/group). **(b)** Heatmap of metabolite levels (*n* = 3/group), with differentially expressed metabolites plotted on the vertical axis and their expression levels represented in a green-to-red gradient. **(c)** Enrichment analysis (*n* = 3/group). The left panel shows significant associations of biological pathways/functions between KO and KO + MA-5 2 mg/kg, while the right panel shows significant associations between KO and KO + MA-5 4 mg/kg. **(d)** Schematic representation of the tricarboxylic acid (TCA) cycle and mitochondrial electron transport chain. **(e)** Comparison of fumarate, pyruvate, and lactate levels in untreated and MA-5-treated cochleae (*n* = 3/group). *p < 0.05. MA-5, mitochonic acid 5; cdk5rap1, cyclin-dependent kinase (CDK)5 regulatory subunit-associated protein 1; KO, knockout; PCA, principal component analysis.

Metabolomic analysis using PCA and heatmap analysis demonstrated distinct metabolic profiles between untreated and MA-5-treated mice (Figures 6a,b). Enrichment analysis indicated that mitochondrial metabolic pathways, including glycogenesis, nucleotide and carbohydrate metabolisms, as well as lipid *β*-oxidation, were activated in MA-5-treated groups (Figure 6c). Notably, pyruvate and lactate levels were decreased in the low-concentration MA-5-treated group, with lactate levels being significantly lower than those in untreated mice (Figure 6d,e*, P* = 0.04; F (2, 12) = 10.1, *p* = 0.03). The high-concentration MA-5-treated group also showed a trend toward lactate suppression (Figures 6d,e).

## 4 Discussion

In this study, we demonstrated that administering MA-5 to C57BL/6 mice with mitochondrial dysfunction and ATP depletion in the inner ear, caused by a deficiency in CDK5RAP1-mediated mt-tRNA chemical modifications, effectively mitigated both cochlear aging and ARHL progression. MA-5 treatment protected cochlear tissue by preventing abnormal mitochondrial morphology in type II and IV fibroblasts of the helical ligament and preserving the intracochlear potential, a critical function of the helical ligament essential for auditory function. Additionally, our findings suggest that MA-5 may prevent aging in helical ligament fibroblasts by facilitating the nuclear translocation of YAP protein, which in turn upregulates SIRT1, a key regulator of the aging process (Zhao and Tian, 2022). However, we also found that high concentrations of MA-5 can induce cochlear injury.

Previous research from our group showed that mitochondrial protein homeostasis disruption and oxidative stress in SLi can accelerate ARHL in CDK5RAP1-deficient mice (Miwa et al., 2022, 2023). The deterioration of mitochondrial quality in these mice was closely associated with an ionic imbalance in the inner ear, which likely contributes to loss of HC and SGC during aging (Miwa et al., 2022). Moreover, degeneration of supporting cells in the SLi has been identified as a key factor in ARHL progression, further emphasizing the importance of maintaining mitochondrial integrity in these structures (Hequembourg and Liberman, 2001).

Our current findings suggest that MA-5 preserved the cochlear potential by preventing the destruction of cristae in mitochondria of type-II and -IV fiber cells in SLi while also preventing Cx26 downregulation in type-I fiber cells. Such preservation is essential for maintaining auditory perception and ion homeostasis in the endolymph, the extracellular fluid critical for sound transmission (Hequembourg and Liberman, 2001). In previous studies, chronic reductions in the endolymphatic potential—caused by genetic variations such as those in the *Brn4* allele—led to increased ABR thresholds (Phippard et al., 1999). However, in the present study, MA-5 effectively preserved the EP and, consequently, the hearing thresholds.

We also conducted metabolomic analyses to assess whether MA-5 could suppress mitochondrial dysfunction, which provided further evidence of MA-5’s protective effects against mitochondrial dysfunction in CDK5RAP1-deficient mice. Although fumaric acid levels did not differ between untreated and MA-5-treated CDK5RAP1-deficient mice, the observed reductions in pyruvate and lactate levels indicated an overall recovery of mitochondrial function, consistent with earlier research (Wasserman et al., 1985). These findings were supported by TEM images, which showed reduced mitochondrial damage—specifically, less cristae loss and mitochondrial swelling—in SLi fibroblasts from MA-5-treated mice, suggests that MA-5 protects against mitochondrial dysfunction (Miwa et al., 2023).

MA-5 is a novel compound that enhances mitochondrial function by stabilizing mitochondrial membranes and improving ATP production. Its potential clinical significance lies in its ability to directly mitigate mitochondrial dysfunction, a key pathological mechanism in several chronic diseases, including ARHL. Considering that ARHL is driven by progressive degeneration of cochlear cells due to mitochondrial damage and oxidative stress, long-term administration of MA-5 may preserve cochlear function by maintaining cellular energy balance and reducing apoptosis. Furthermore, ARHL has been associated with cognitive decline and increased risk of dementia, suggesting that preventing or delaying its progression may have broader implications for brain health in aging populations. In preclinical models, MA-5 has shown promise in reducing cochlear cell damage, underscoring its potential as a preventive or therapeutic agent. Long-term MA-5 administration could offer a novel, non-invasive approach to ARHL management, potentially improving quality of life and reducing healthcare burdens associated with hearing loss and cognitive impairment.

Previous reports suggest that MA-5 can enhance mitophagy via the MAPK–YAP signaling pathway, promoting the clearance of damaged mitochondria (Lei et al., 2018). Additionally, YAP maintains SIRT1 activity, which is crucial for aging, and enhances mitofusin 2 (Mfn2) expression, further supporting mitochondrial integrity (Yan et al., 2018). Both YAP and SIRT1 levels decline with physiological aging, as observed in skin stromal cells (Sladitschek-Martens et al., 2022) and cochlear tissue (Takumida et al., 2015). Notably, MA-5 prevented the reduction of cochlear SIRT1 expression, suggesting that it may inhibit cochlear aging by maintaining mitochondrial function through the Hippo–YAP signaling pathway (Lei et al., 2018; Yan et al., 2018). The results of our study further support this model, as MA-5 promoted nuclear YAP translocation in helical ligament fibroblasts, a process that can activate SIRT1 and likely help preserve cochlear function.

This study has some limitations. First, higher doses of MA-5 were associated with intracochlear injury, including increased shedding of OHCs. Although no studies have previously reported tissue injury from high doses of MA-5, Xin et al. demonstrated that mitochondrial function enhancing effect is reduced at high doses. To optimize the therapeutic potential of MA-5, future experiments should include additional dose settings to confirm efficacy and identify the optimal therapeutic range. Pharmacokinetic analysis are also necessary to determine the correlation between MA-5 concentrations in blood and tissue with its effectiveness. Additionally, further investigation is required to understand the mechanisms underlying the reduced efficacy at high doses, particularly by evaluating mitochondrial function and cellular stress markers. Since we did not assess the precise localization of MA-5 within the cochlear tissues, it is possible that a localized high MA-5 concentration contributed to tissue damage. Future studies should investigate the distribution of MA-5 within the cochlea to develop strategies for mitigating such effects. The second limitation is that the CDK5RAP1-deficient mouse model used in this study may not fully replicate human ARHL, particularly because it does not exhibit SV atrophy, which is a hallmark of human ARHL. Therefore, additional models or genetically modified mice may be necessary to gain more comprehensive understanding of MA-5’s effects. Third, while we suggested that MA-5 may enhance mitochondrial function through “2-methylthiolation of mitochondrial transfer RNA,” there is currently no direct evidence supporting this mechanism. Future research should aim to clarify whether MA-5 directly influences 2-methylthiolation of mitochondrial transfer RNA, and how it contributes to mitochondrial homeostasis. Fourth, a notable limitation of our study was the lack of complete concordance among the results of ABR, DPOAE, EP measurements, SGCs morphology, and spiral ganglion neuron function. These discrepancies may have arisen because EP was recorded at the middle turn of the cochlea during cochlear puncture, leading to variations across different regions of the cochlea. Moreover, while chronic reductions in EP can induce progressive tissue damage, transient reductions are less likely to result in structural impairment. This difference in pathological progression may explain some of the observed outcomes. Moving forward, optimizing EP measurement techniques and refining the experimental design will be critical for enhancing the validity and reproducibility of the findings. Finally, the study lacked a control group consisting of WT mice treated with MA-5. Without this comparison, it remains unclear whether MA-5 has protective effects against age-related cochlear degeneration in otherwise healthy mice. Including a WT MA-5 treated group in future experiments would help determine the preventive effect of MA-5 against ARHL in non-pathological aging.

## 5 Conclusion

Our findings highlight the potential of MA-5 in preventing mitochondrial quality deterioration and the associated disruption of ion-transport systems, particularly in SLi fibrocytes, which are vulnerable to age-related mitochondrial dysfunction and accumulation of mtDNA mutations. By activating the Hippo-YAP signaling pathway, MA-5 might inhibit SLi cell aging, preserve the mitochondrial integrity, and prevent a chronic declines in the EP, thereby mitigating HC degeneration and the secondary loss of SGC. These findings suggest that MA-5 may be a viable treatment option for ARHL associated with mitochondrial dysfunction, particularly in cases associates with CDK5RAP1 deficiency and its impact on mt-tRNA modification. Further studies are warranted to assess the efficacy of MA-5 in additional ARHL models to confirm its broader therapeutic applicability.

## Data Availability

The original contributions presented in the study are included in the article/Supplementary material, further inquiries can be directed to the corresponding author.

## Footnotes

^1^ https://www.metaboanalyst.ca/

^2^ https://spatial-model-editor.github.io/
