# Supplementary material for: Mitochonic acid 5 mitigates age-related hearing loss progression by targeting defective 2-methylthiolation in mitochondrial transfer RNAs
Source: Front Cell Neurosci. 2025 Apr 7;19:1541347. doi: 10.3389/fncel.2025.1541347 (PMC12009901; doi:10.3389/fncel.2025.1541347)
Supplement: Supplementary file 1 [file Table_1.DOCX]

Supplementary Material

# Supplementary Figures


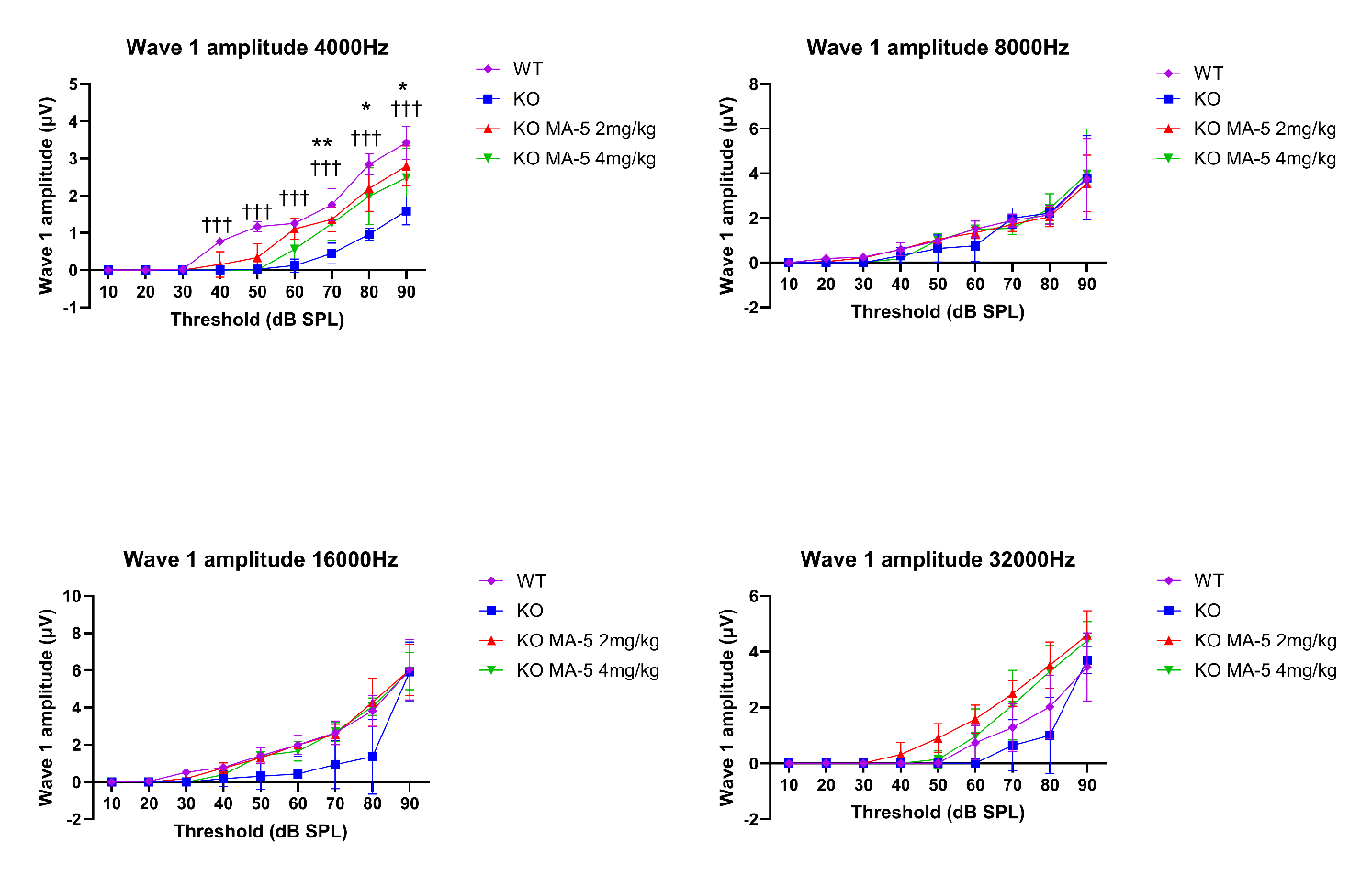


**Supplementary Figure 1.** **ABR wave I amplitude level curves in MA-5-treated *Cdk5rap1*-KO mice.**

ABR wave I amplitude levels in *Cdk5rap1*-KO untreated mice and WT untreated mice at 20 weeks of age, compared to those in MA-5-treated mice across various frequencies (n = 5 mice/group). Error bars represent the mean ± SE of five independent experiments. ^†††^ indicates significant differences (*p* < 0.001) between WT and untreated KO mice. * indicates significant differences (**p* < 0.05, ***p* < 0.01) between untreated KO mice and MA-5-treated mice (2 mg/kg).

*Abbreviations*: MA-5, mitochonic acid 5; cdk5rap1, cyclin-dependent kinase (CDK)5 regulatory subunit-associated protein 1; KO, knockout; WT, wild-type; ABR, auditory brainstem response.


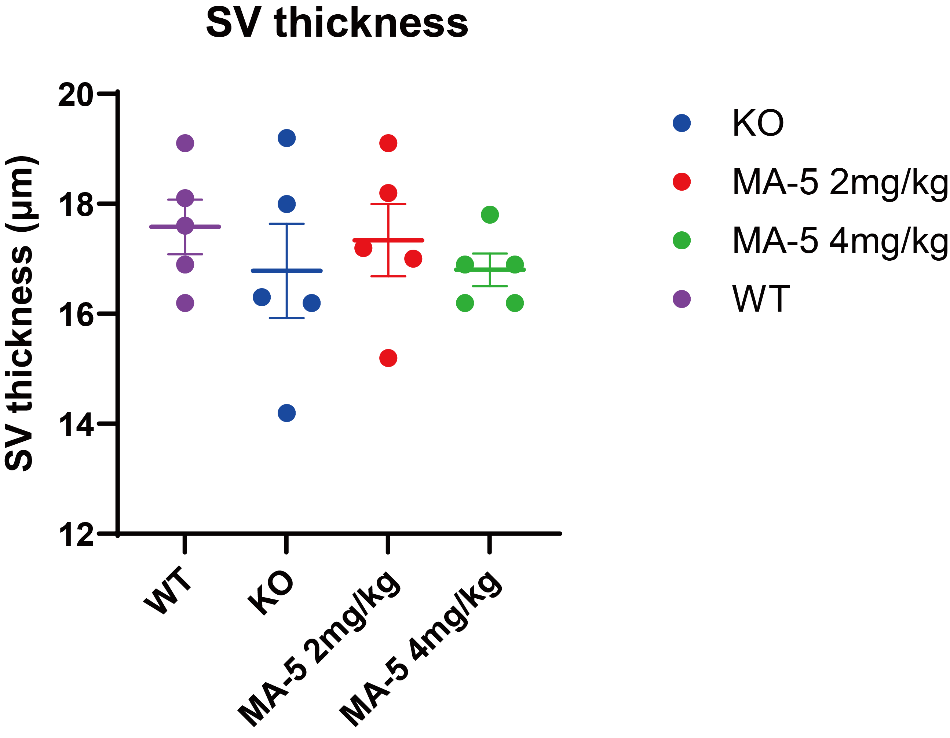


**Supplementary Figure 2.** **SV thickness in MA-5-treated *Cdk5rap1*-KO cochleae.**

*Abbreviations*: MA-5, mitochonic acid 5; cdk5rap1, cyclin-dependent kinase (CDK)5 regulatory subunit-associated protein 1; KO, knockout; SV, stria vascularis

# Supplementary Tables

**Supplementary Table 1. Number of animals used for treatment and post-mortem experiments**

| Assessment | n |
| --- | --- |
| KO DMSO or KO MA-5 2mg/kg, KO MA-5 4mg/kg (each n = 33) | |
| ABR (Threshold, Wave I), DPOAE→H&E (Stria vascularis thickness) | 5 |
| EP | 5 |
| Cryostat→IHC (Tuj1[Spiral ganglion cells count]), Na^+^/K^+^-ATPase α1 [mLI], Cx26 [mLI], SIRT1 [mLI], YAP [mLI and Nuclear:cytoplasmic ratio calculations]) or SA-βgal | 5 |
| TEM | 5 |
| Surface prep (Hair cell count) | 5 |
| ELISA (SIRT1), Western blot (YAP) | 5 |
| Metabolome analysis | 3 |
| WT DMSO (n = 25) | |
| ABR (Threshold, Wave I), DPOAE→H&E (Stria vascularis thickness) | 5 |
| EP | 5 |
| Cryostat→IHC (Tuj1[Spiral ganglion cells count], Na^+^/K^+^-ATPase α1[mLI], Cx26[mLI], SIRT1[mLI], YAP[mLI and Nuclear:cytoplasmic ratio calculations]) or SA-βgal | 5 |
| TEM | 5 |
| Surface prep (Hair cell count) | 5 |

*Abbreviations*: KO, knockout; MA-5, mitochonic acid 5; ABR, auditory brainstem response; DPOAE, distortion product otoacoustic emission; H&E, hematoxylin and eosin staining; EP, endocochlear potential; IHC, immunohistochemical staining; Cx26, connexin 26; mLI, modified labeling index; TEM, transmission electron microscopy; WT, wild-type; DMSO, dimethyl sulfoxide; Tuj1, beta-tubulin III; SIRT1, sirtuin 1; YAP, yes-associated protein; ELISA, enzyme-linked immunosorbent assay; SA-βgal, senescence-associated β-galactosidase.
